# Self-regulation therapy increases frontal gray matter in children with fetal alcohol spectrum disorder: evaluation by voxel-based morphometry

**DOI:** 10.3389/fnhum.2015.00108

**Published:** 2015-03-04

**Authors:** Debra W. Soh, Jovanka Skocic, Kelly Nash, Sara Stevens, Gary R. Turner, Joanne Rovet

**Affiliations:** ^1^Department of Psychology, York UniversityToronto, ON, Canada; ^2^Neurosciences and Mental Health Program, The Hospital for Sick ChildrenToronto, ON, Canada; ^3^Department of Applied Psychology and Human Development, The Ontario Institute of Studies in Education, University of TorontoON, Canada; ^4^Department of Psychology, University of TorontoON, Canada; ^5^Department of Pediatrics, University of TorontoON, Canada

**Keywords:** FASD, executive functioning, self-regulation of emotions, neuroplasticity, VBM, Alert therapy

## Abstract

Children with fetal alcohol spectrum disorder show executive function (EF) deficits, particularly in self-regulation skills, and abnormalities in brain regions critical for these skills. None of the validated EF interventions for these children has been evaluated with regards to impacts on brain structure. Twenty-nine children with FASD were assigned to either an immediate-treatment (TX) or delayed-treatment control (DTC) group (DTC). Nineteen typically developing children served as healthy controls (CT). All received a structural MRI scan and baseline neuropsychological testing, following which the TX group underwent 12 weekly 1.5-h sessions of the Alert Program for Self-Regulation^®^. After treatment or a period of ~14 weeks, all received a repeat scan and post-intervention testing. Whole-brain and region-of-interest analyses using voxel-based morphometry evaluated group differences and changes over time in gray matter (GM). Exploratory analyses revealed significant group changes: (1) At baseline, combined TX and DTC groups demonstrated global GM reductions compared with the CT group. (2) Region-of-interest analysis using a frontal mask, comparing post-intervention to pre-intervention results, showed significantly increased GM in the left middle frontal gyrus (BA10), right frontal pole (BA11), and right anterior cingulate (BA32) in the TX group. Similar results were not found in the DTC or CT groups. (3) At post-intervention, both TX and CT groups showed larger GM volumes than the DTC group in the left superior frontal gyrus (BA9), which was smaller in the FASD group at baseline. These results suggested that Alert led to improvements in post-intervention testing of self-regulation skills and typical brain development in treated children.

## Introduction

Fetal Alcohol Spectrum Disorder (FASD) is the umbrella term used to describe the often challenging developmental abnormalities that arise from prenatal alcohol exposure (PAE) and affects as many as 4% of children in North America (May et al., 2014). The three primary forms of FASD are Fetal Alcohol Syndrome (FAS), partial Fetal Alcohol Syndrome (pFAS), and Alcohol-Related Neurodevelopmental Disorder (ARND). FAS, which is characterized by the symptom triad of a distinctive dysmorphic face (i.e., small palpebral fissures, elongated philtrum, thin vermillion), growth abnormalities, and cognitive and behavioral impairments (Stoler and Holmes, 1999), is generally considered the most severe disorder along the fetal alcohol spectrum; pFAS with fewer or less severe physical features is considered a milder variant (Stratton et al., 1996). ARND, which involves only the cognitive and behavioral sequelae, is the most common FASD subtype (Chudley et al., 2005). All forms are associated with a characteristic profile of cognitive and socioemotional disturbances (Steinhausen et al., 1982; Fryer et al., 2007), especially attention deficit hyperactivity disorder (ADHD) (Nanson and Hickcock, 1990; Coles et al., 1997; Bhatara et al., 2006) and conduct disorder (Nash et al., 2011). Most adults with FASD evince later mental health problems (Streissguth et al., 2004), particularly depression and substance abuse, while many also experience trouble with the law (Fast and Conry, 2009). Not surprisingly, FASD places an extraordinary burden on parents or caregivers (Leenaars et al., 2012), as well as teachers, and it results in a high cost to society (Lupton et al., 2004; Stade et al., 2007).

The cognitive deficits seen in children and adolescents with FASD include difficulties in language, visuospatial, attention, and memory domains (Ramussen et al., 2006; Mattson et al., 2011; Nash et al., 2013). However, their most striking impairment is their deficit in executive functioning (EF; Kodituwakku, 2007). This is thought to reflect a core and intractable feature of the condition (Kodituwakku et al., 1995) that persists throughout life (Connor et al., 2000). One key and especially problematic aspect of their EF dysfunction that is evident from birth is their difficulty with self-regulation (Kodituwakku, 2010), and more specifically, their inability to stay alert and focused, control impulses, and regulate their emotions.

In recent years, a number of neuroimaging studies on individuals with FASD have served to identify some of the neuroanatomical abnormalities contributing to their primary cognitive problems (Spadoni et al., 2007; Norman et al., 2009). For example, Fryer et al. (2007) showed that the frontal-striatal network, which is critical for EF, functions abnormally in children with FASD. Also reported is a reduced size of orbitofrontal regions (Spadoni et al., 2007; Lebel et al., 2011), which are implicated in impulse control (Stevens and Haney-Caron, 2012). Furthermore, a recent study using voxel-based morphometry (VBM) has shown dose-dependent reductions between gray and white matter volumes of both subcortical and cortical brain regions and levels of PAE (Eckstrand et al., 2012). The findings from this study suggest that VBM may be a promising tool for assessing brain changes in FASD.

From a different perspective, an emerging literature has attempted to identify effective interventions for preventing the adverse consequences of FASD, given findings that adults with PAE had fewer secondary disabilities if they were diagnosed (and presumably treated) very early in life (Streissguth et al., 2004). Surprisingly, however, few empirically-validated therapies exist for this population and of those described, results are difficult to interpret due to large age ranges (Bertrand, 2009; Peadon et al., 2009) and very small sample sizes (Timler et al., 2005). Furthermore, core deficits, such as poor self-regulation, have seldom been addressed (Kodituwakku and Kodituwakku, 2011). Additionally, studies evaluating the neuroplastic changes associated with these interventions have not been conducted, despite claims this represents an ideal approach for evaluating true treatment effects (Stuss and Levine, 2002). Although VBM has been used to measure the success of treatments such as cognitive-behavioral therapy and pharmacotherapy in other pediatric populations (e.g., obsessive-compulsive disorder; Lazaro et al., 2009; Huyser et al., 2013), this has not yet been attempted in children with FASD.

Given the primacy of self-regulation difficulties in children with FASD, a therapeutic approach targeting these skills holds promise. One such therapy is the Alert Program for Self-Regulation® (Williams and Shellenberger, 1996), which is a 12-week manualized program focusing on sensory integration and cognitive behavioral training. Research in a variety of other clinical populations shows Alert is associated with improved self-regulation skills in children. For example, children with emotional disturbances given Alert later showed reduced aggression (Barnes et al., 2008). To date, two studies have used the Alert program in children with FASD (Wells et al., 2012; Nash et al., 2015). The results have shown improved social problem-solving and inhibitory control skills. However, the neuroplastic changes associated with these behavioral improvements have not been examined.

The current study therefore evaluated whether children with FASD who receive the Alert program will show neuroanatomic changes in the brain regions that underlie self-regulation. In this study, we used VBM to compare frontal gray matter volume changes of children with FASD treated with Alert, versus. those waitlisted to receive Alert upon study completion, and typically developing controls. Our goal was to determine whether the neuroanatomy of the FASD group following Alert more closely resembles that of typically developing control children than the waitlist group. We hypothesized that: (1) all children with FASD at baseline will show structural brain abnormalities in frontal regions critical for self-regulation, specifically the orbital, ventromedial, and dorsolateral prefrontal cortices (Davidson, 2002) and anterior cingulate; (2) on rescanning, children with FASD in the Alert-treated group will show structural gray matter volume changes in these regions while those in the waitlist condition will not show these changes; and (3) following Alert, the frontal neuroanatomy of children with FASD will be similar to controls but not children with FASD assigned to the waitlist condition. As a supplementary analysis, we explored whether treatment-related structural changes were associated with behavioral improvements in emotion regulation and inhibitory control.

## Materials and methods

### Participants

A total of 65 children aged 8–12 years were originally recruited for this study, 38 with FASD and 27 typically developing controls (CT). The FASD group was ascertained through the Motherisk Clinic at the Hospital for Sick Children (SickKids), where cases were identified from clinic files or from Ontario-based FASD support groups. Parents/caregivers of Motherisk cases were sent a letter describing the project while the remainder was notified of the project via postings at local FASD support groups or through the FASworld regional network. Families from these sources were asked to contact our project coordinator, who conducted a telephone-screening interview to assess eligibility.

The diagnostic process for Motherisk-derived cases involved a thorough assessment conducted by a team comprised of a pediatrician, psychologist, psychometrist, and speech therapist, who used the Canadian Diagnostic Guidelines system (Chudley et al., 2005) to formulate a diagnosis; the majority of other cases were diagnosed at an accredited FASD diagnostic facility throughout Ontario that used the Washington 4-digit code system (Astley and Clarren, 2000). Essential for diagnosis at both sources was documented or first-hand evidence (e.g., from a biological relative) of excessive maternal alcohol consumption during pregnancy and significant neuropsychological deficits in a minimum of three specified domains. Two of the casses derived through community postings were lacking a formal FASD evaluation. However, the adoptive relative (grandmother, aunt) claimed to have seen mother drink excessively throughout pregnancy while the child's currently described array of severe cognitive and behavior problems are concordant with an FASD diagnosis, based on the senior investigator's (JR) clinical expertise with this population. Because the Motherisk Clinic did not originally use the pFAS designation, all children diagnosed at this source were determined to have ARND, as none had FAS. The final sample therefore consisted of 22 children with ARND, 4 with pFAS, 1 with FAS, and two with an unspecified subtype since formal evaluations were not performed on them.

The typically developing control sample was recruited through community and hospital postings, or included biological or non-exposed adopted children of a participating foster or adoptive parent. All of these children were screened via telephone interview with the mother to ensure the child was not prenatally exposed to alcohol or other teratogenic substances and did not have a psychiatric diagnosis (e.g., ADHD) or learning disability.

Across groups, exclusionary criteria at time of study entry were a head injury requiring hospitalization, other neurological abnormalities, a debilitating or chronic medical condition, and contraindications to MRI (e.g., braces; other implanted metal devices). Unfortunately, a few cases began wearing braces between pretest and posttest sessions.

### Design

Upon study enrollment, children in the FASD group were randomly assigned to either an immediate treatment (TX) group (*n* = 20) or a waitlist delayed-treatment control (DTC) group (*n* = 18). The only exceptions were children from the same family, who were assigned to the same group for the family's convenience. This happened in four families: two with siblings assigned to the TX group and two assigned to the DTC group. One family assigned to the TX group requested reassignment to DTC after pretest, as they were unavailable to attend therapy in the said period.

The design included baseline testing and scanning (pretest), treatment of the TX group, retesting and rescanning (posttest), and treatment of the DTC group. All study-related activities took place at SickKids.

### Procedures

The baseline (pretest) assessment took place over a 2-day period separated by 2 to 30 days, in order to accommodate families' schedules. On the first day of pretest, children received a broad test battery that included the Wechsler Abbreviated Scale of Intelligence (Wechsler, 1999) and selected subtests from the NEPSY-II (Korkman et al., 2007), as well as tests of social cognition and empathy (Stevens et al., 2015). Parents also completed several questionnaires while the child was being tested. These included a child-history questionnaire from which information on child's background, psychopathology and related treatment were ascertained and the Behavior Rating Inventory of Executive Function (BRIEF) (Gioia et al., 2000) and Child Behavior Checklist (Achenbach, 2001). For current purposes, only two indices were used: the Emotional Control subscale of the BRIEF, which assesses child's ability to modulate emotional responses appropriately in daily functioning, and the NEPSY-II Inhibition subtest score, which measures child's ability to inhibit an automatic response in favor of a novel one. These indices were chosen following preliminary statistical analyses showing they produced the largest effect sizes in differentiating the groups (Nash et al., 2015). On the second pretest day, all children underwent a 1-h MRI scanning session that included both structural and functional (reported elsewhere) MRI sequences.

Retesting and rescanning (posttest) took place on a single day approximately 2 weeks after Alert was finished (TX group) or 14 weeks after baseline (DTC and CT groups). The posttest battery was shorter than pretest because not all tests were repeated; the posttest scan was the same as at pretest. Shortly after posttest, the DTC group commenced therapy. Within 1 month of the first scan, children's physicians were sent copies of the neuroradiological report. Approximately 2 months after posttest, parents/caregivers received a report describing their child's results. Parent/caregivers of children with FASD were also invited back for a 1-h feedback session to discuss the report and modes of implementing the therapeutic tools at home and school. Families were compensated for all travel expenses, including overnight stays.

All procedures were approved by the Research Ethics Boards at SickKids and the University of Toronto. This study was registered with the Canadian Clinical Trials Protocol Registration and Results System.

### Therapy

Approximately 1 week after pretest scanning, the TX group started Alert therapy (Williams and Shellenberger, 1996; Wells et al., 2012). This 12-week program provides 1.5-h sessions of sensory integration and cognitive behavioral training. The approach is based on the analogy of a car engine that runs at different speeds. Children learn to recognize when their engines are running too quickly or too slowly and modulate their behavior accordingly to allow their engines to run “just right.” The program is provided in three stages: Stage 1 trains children to label their engine levels and become familiarized with them; Stage 2 gives children strategies for changing their engine speeds; Stage 3 teaches them to choose among strategies and apply them in real-life situations. Stage progression is dependent on mastery of the stage prior.

Therapy was individually administered to the children by either a therapist or one of two doctoral-level clinical psychology students, all of whom were formally trained by Alert program developers. The therapy took place in a quiet room devoid of unnecessary distractions and containing floor mats, therapy balls, inner tubes, pillows, tent, caterpillar tunnel, and manipulanda, as specified by the Alert program. Although we aimed to complete each child's therapy within 14 weeks, the period was longer in some cases due to illnesses, holiday interruptions, therapist unavailability, school events, and extraordinary circumstances (e.g., car failure). It should be noted that some families drove as far as 150 miles each way to attend weekly therapy sessions, necessitating the overnight housing of some families in a nearby hotel.

### Image acquisition and processing

MRI data were collected on a GE Medical Systems Signa Excite 1.5T MRI scanner (GE Healthcare, Milwaukee, WI) in the Diagnostic Imaging Unit at SickKids. Images were acquired using a T1-weighted inversion recovery prepared fast spoiled gradient echo image (repetition time = 10.372 ms; echo time = 4.264 ms; inversion time = 400 ms; flip angle = 20°; field of view of 24 cm; in-plane voxel dimensions were 0.9375 × 0.9375 mm with slice thickness of 1.5 mm reconstructed at 1 mm^3^).

All scans were processed using VBM Toolbox v5.1 (http://dbm.neuro.uni-jena.de/vbm) in Statistical Parametric Mapping 5 (SPM5; Wellcome Department of Imaging Neuroscience, University College London, UK; http://www.fil.ion.ucl.ac.uk) running on MATLAB 7.3.0 R2006b (MathWorks, Natick, MA). Briefly, preprocessing included aligning all scans to the anterior commissure, normalization to a standard Montreal Neurological Institute (MNI) template, bias-correction, segmentation into gray and white matter and CSF using the International Consortium for Brain Mapping (ICBM) tissue probability maps, and smoothing of segmented images using a 8 mm Gaussian kernel. Total gray matter, total white matter, and CSF volumes were extracted from the segmented files. Gray matter was analyzed via the default VBM 5.1 parameters using both a whole-brain and a region-of-interest (ROI) approach to limit the voxels included in analyses. For the *a priori* ROI analysis, we used an explicit mask based on the anatomically-based template of Kabani and colleagues (Kabani et al., 2002; Mabbott et al., 2009). Briefly, this mask divides the brain into eight cerebral regions, namely frontal, parietal, temporal, and occipital lobes of both hemispheres. We currently used the bilateral frontal cortical regions portion.

### Statistical approach

For demographic and behavioral data, between-group differences were analyzed via *t*-tests and chi-square tests in SPSS 22.0 (IBM Corp., 2013). To assess changes in behavior on primary indices of emotion regulation and inhibition, repeated measures group X session ANOVAs were conducted and η^2^ values were obtained. MRI data were analyzed using pairwise two-tailed *t*-tests in SPM5 to evaluate between-group (e.g., TX vs. DTC; TX vs. CT; CT vs. FASD) differences in gray matter at posttest and pretest sessions. Within each group, repeated measures ANOVAs served to assess volume changes between pretest and posttest sessions. A minimum contiguous cluster size threshold of 5 voxels was used (Worsley et al., 1999). Covariates of no interest were: (1) intracranial volume (ICV), based on total gray matter, white matter, and CSF; (2) age, given the subtle changes in gray matter volumes known to occur within the age range currently studied (Giedd, 2008); and (3) IQ, given our earlier finding that IQ was significantly lower in FASD than control groups (Greenbaum et al., 2009).

To address Hypothesis 1, we first compared the total FASD group and controls at baseline (pretest) using two-tailed *t*-tests; both whole-brain and region-of-interest analyses were conducted on these data. To address Hypothesis 2, we used a repeated-measures design to identify the changes in neuroanatomy that occurred between sessions in each of the groups. To address Hypothesis 3, we conducted pairwise comparisons between TX and DTC and TX and CT groups at posttest in order to discern whether the TX group's neuroanatomy now closely resembled that of the CT group, and differed from the DTC group. To explore the associations between changes in brain structure and changes in behavior, we performed in SPM5 simple correlations between posttest gray matter findings and posttest change scores for the BRIEF Emotional Control subscale and NEPSY-II Inhibit measures.

## Results

### Demographics and behavioral results

**Figure 1:**
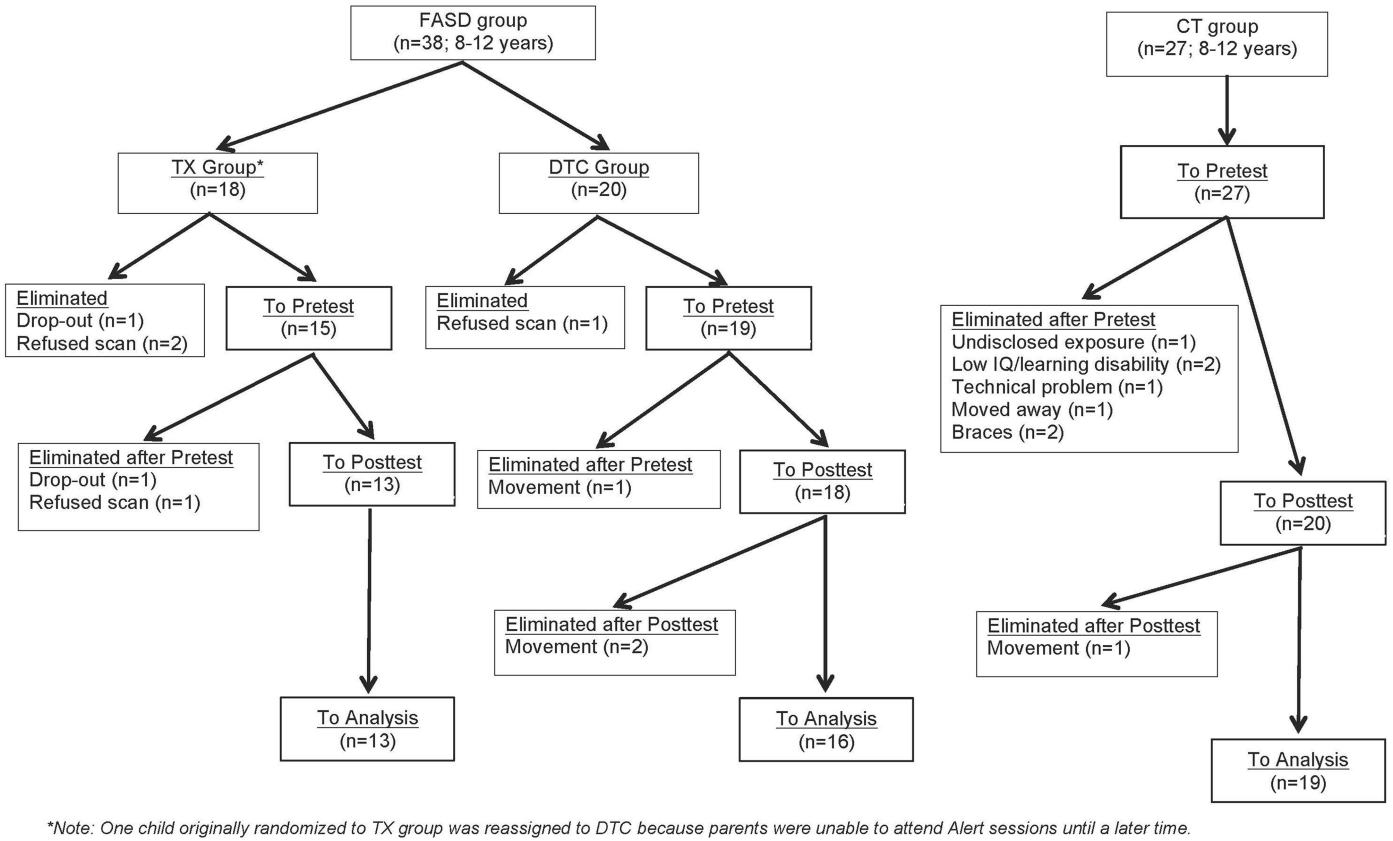
**CONSORT flow diagram of progress through enrolment, pretest, posttest, and data analysis for FASD and CT groups**.

Figure 1 provides a CONSORT diagram of the flow of cases. Of the 65 originally recruited, 17 were eliminated for the following reasons: One child with FASD dropped out before ever coming to hospital due to court appearances, two children (1 FASD, 1 CT) dropped out or moved before the second scan, two children in the CT group began wearing braces either between scans, four children with FASD refused one or both scans due to anxiety, three CT children were eliminated after the first scan when it was determined they had either an undisclosed learning disability or exposure to another substance during pregnancy, technical problems were incurred during the scan of one CT child, and the scans from four children (3 FASD, 1 CT) were eliminated due to excessive movement in the scanner (i.e., based on visual inspection of images). Of the 9 eliminated FASD cases, 5 belonged to the TX group and 4 to the DTC group, most of whom (75%) were diagnosed at Motherisk. Note that the two FASD subgroups differed with regard to why their scans were not included in analysis, with a greater number of drop-outs and refusals in the TX group, and excessive motion in the DTC group. Participants and excluded cases did not differ on other indices. The final sample therefore consisted of 13 TX (8 ARND, 3 pFAS, 2 unknown), 16 DTC (14 ARND, 1 pFAS, 1 FAS), and 19 CT.

Table 1 presents the demographic data of the final sample contributing analyzable pretest and posttest scans. Groups did not differ in age, sex, or handedness. However, the CT group scored higher in IQ than both FASD subgroups (*p* < 0.001). The interval between scans was longest in TX (18.6 weeks) followed by DTC (16.7 weeks) and then CT (15.7 weeks, *p* < 0.01). Within the FASD sample with good scans, a greater proportion cases recruited through Motherisk belonged to the DTC subgroup while the proportion of those diagnosed elsewhere was larger in TX; however, the differences were not significant (χ^2^ = 2.43, *p* > 0.05). This likely reflected the greater loss of Motherisk-recruited cases within the TX subgroup. The two FASD subgroups did not differ in incidence of ADHD comorbidity or particular type of FASD classifications, even though the incidence of ARND appeared to be higher in DTC and pFAS higher in TX.

**Table 1:** **Demographic Characteristics of the FASD and Control Groups**.

**Variables**	**TX (*n* = 13)**	**DTC (*n* = 16)**	**CT (*n* = 19)**	***p*-value**	**Significant effects**
Age (years)	9.46	9.88	10.05	n.s.	
Sex (# males)	6	7	11	n.s.	
Handedness (% right)	84.6	87.5	94.6	n.s.	
FASD Classifications				n.s.	
FAS	0	1	N/A		
pFAS	3	1	N/A		
ARND	8	14	N/A		
FASD diagnosis unknown	2	0	N/A		
Recruitment source^a^					
Motherisk	1	5	N/A		
Other	12	11	N/A		
ADHD Comorbidity	7	9	0	0.001	TX, DTC>CT
IQ	84.46	93.81	112.42	0.001	TX, DTC<CT
Interval between scans (weeks)	130.2	116.6	109.7	0.002	TX>DTC>CT

*TX, Alert-treated FASD group; DTC, delayed-treatment control FASD group; CT, typically developing control group*.

Table 2 presents the behavioral results for the two indices most strongly differentiating groups. For the BRIEF Emotional Control scale, ANOVA revealed significant main effects of group, *F*_(2, 58)_ = 68.30, *p* < 0.001, and time, *F*_(1, 58)_ = 10.93, *p* = 0.002, as well a significant group X time interaction, *F*_(2, 58)_ = 6.49, *p* = 0.003. These findings reflected the largest posttest improvement by TX group (Table 2). For the NEPSY-II Inhibition subtest, results yielded a significant main effect of group, *F*_(2, 58)_ = 13.96, *p* < 0.001, and group X time interaction, *F*_(2, 58)_ = 4.93, *p* = 0.011. These reflected the superior performance of CT to TX and DTC groups and the greater gains at posttest by TX and CT groups than the DTC group, whose scores declined at posttest.

**Table 2:** **Descriptive statistics for BRIEF and NEPSY-II scores**.

	**TX**	**DTC**	**CT**	***p*-value**	***Comparison***
**BRIEF EMOTIONAL CONTROL^a^**					
Pre-test	73.22±10.6	79.11±5.6	49.54±10.6	0.000	TX, DTC>CT
Post-test	64.97±12.6	79.95±7.3	47.03±10.1	0.000	DTC>TX>CT
Post – Pre	−8.25±3.3	−0.84±8.7	−2.52±7.1	0.04	TX>CT>DTC
**NEPSY-II INHIBITION^b^**					
Pre-test	6.29±3.3	7.27±3.4	9.58±2.2	0.002	CT>TX, DTC
Post-test	7.22±3.7	5.94±3.1	11.08±2.5	0.000	CT>TX, DTC
Post – Pre	0.93±3.2	−1.33±3.3	1.50±2.7	0.01	CT, TX>DTC

a *A lower score signifies better control and a large negative difference score signifies greater gain, M = 50, SD = 10*.

b *A higher score signifies better performance and a large positive difference score signifies greater gain, M = 10, SD = 3. Note: TX, Alert-treated FASD group; DTC, delayed-treatment control FASD group; CT, typically developing control group*.

### VBM results

#### Hypothesis 1

**Figure 2:**
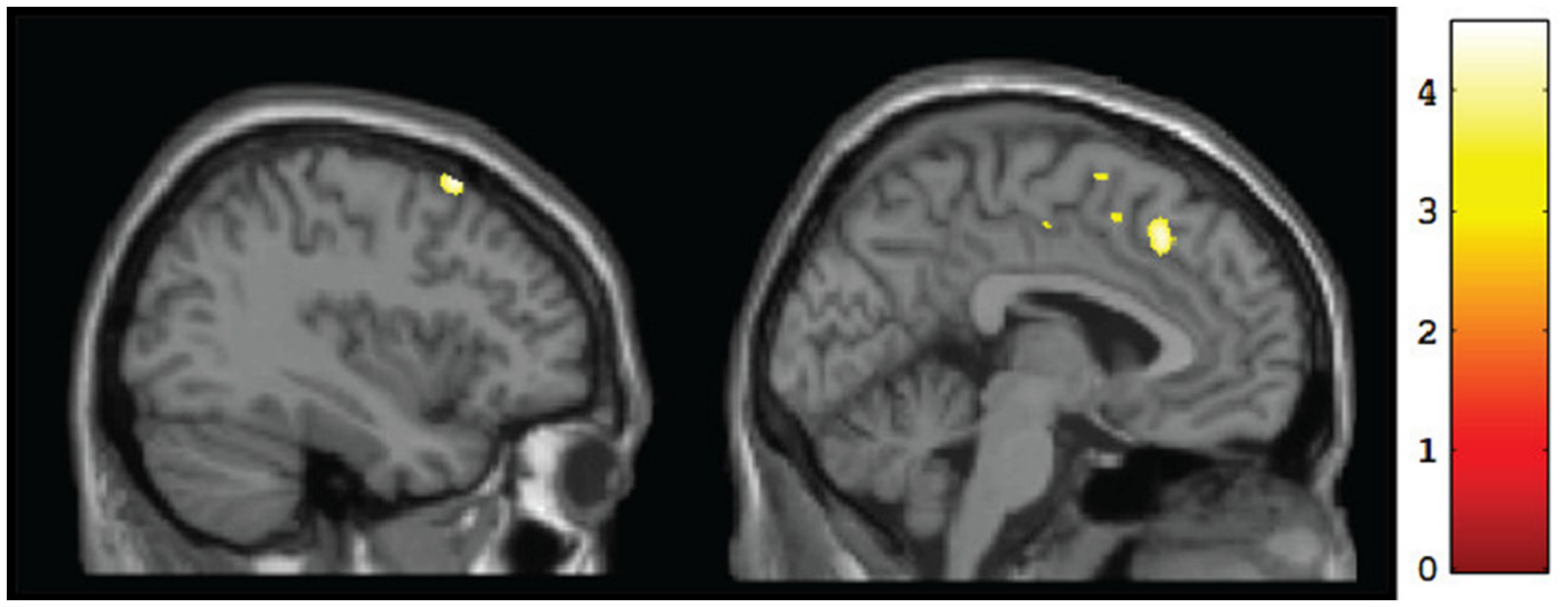
**Sagittal views of regions showing significant (*p* <0.001, uncorrected) differences in gray matter volume at baseline for CT vs. FASD (TX and DTC combined): left superior frontal gyrus (BA8) [−37, 17, 60] (left) and left medial frontal gyrus (BA8) [−3, 25, 44] (right)**.

A whole-brain analysis comparing TX and DTC groups on pretest scans showed no significant differences at both corrected and uncorrected *p*-values. Comparing the CT group with the combined FASD (i.e., TX and DTC) group at pretest, we found no significant between-group differences with the FDR correction applied. However, uncorrected results revealed multiple regions significantly differentiating the groups (*p* < 0.001 level). These results are presented in Table 3 (cluster differences larger than 200 voxels) and Supplementary Table 1 (remaining regions and FASD > CT comparison). For the large cluster effect regions (Table 3), the CT group had larger gray matter volumes than FASD in the bilateral postcentral gyrus (BA2, BA5), left cingulate gyrus (BA24), left superior frontal gyrus (BA8), bilateral medial frontal gyrus (BA8, BA32), left rectal gyrus (BA11), and right inferior parietal lobule (BA40). Using a region-of-interest analysis with a frontal mask applied, we found CT had significantly larger gray matter volumes than FASD (*p* < 0.001, uncorrected) in the left superior frontal gyrus (BA8) and bilateral medial frontal gyrus (see Figure 2).

**Table 3:** **Significant (uncorrected) pairwise comparisons between FASD-combined and control (CT) groups using a whole-brain analysis**.

**Group**	**Region**	**Brodmann area**	**MNI coordinates**			***Z*-statistic**	***p*-value**	**Cluster size**
			***X***	***Y***	***Z***			
CT > FASD	Left postcentral gyrus	2	−50	−28	53	4.66	0.000	450
	Left cingulate gyrus	24	−13	5	38	4.09	0.000	557
	Left superior frontal gyrus	8	−37	17	60	3.97	0.000	211
	Left medial frontal gyrus	8	−3	25	44	3.90	0.000	573
	Right medial frontal gyrus	32	2	8	49	3.65	0.000	221
	Left rectal gyrus	11	−1	20	−28	3.58	0.000	206
	Right postcentral gyrus	5	33	−45	67	3.55	0.000	259
	Right inferior parietal lobule	40	47	−37	61	3.49	0.000	257

*Findings indicate where CT group showed larger gray matter volumes than FASD group. Only clusters larger than 200 voxels are listed*.

#### Hypothesis 2

**Figure 3:**
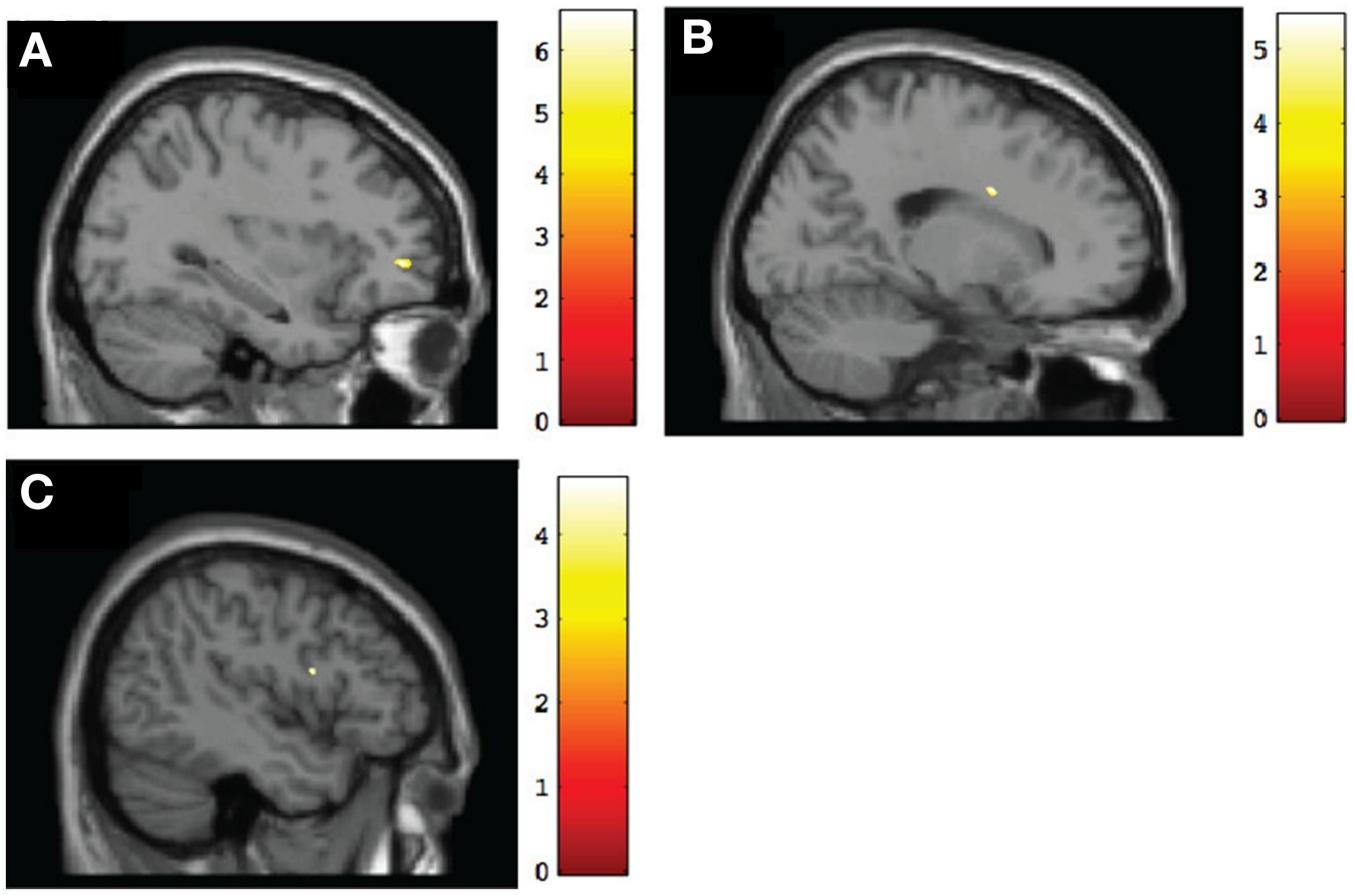
**Sagittal view of regions involving significant within-group increases of gray matter volume at post-intervention. (A)** TX group, left middle frontal gyrus (BA10) [−32, 47, 0], *p* < 0.001, uncorrected; **(B)** DTC group, right cingulate gyrus (BA24) [19, 4, 30], *p* < 0.001, uncorrected; **(C)** CT group, left inferior frontal gyrus (BA9) [−45, 6, 18], *p* < 0.001, uncorrected.

To test for within-group changes between pretest and posttest sessions, we examined only ROI findings with the frontal mask applied. Results with the FDR correction applied found no significant changes in any of the groups. However, when we used uncorrected data, the TX group was observed to have a significant (*p* < 0.001, uncorrected) posttest gray matter volume increase in the left middle frontal gyrus (BA10), right frontal pole (BA11), and right anterior cingulate (BA32) (Table 4; see also Figure 3A). In contrast, the DTC group showed a significant (*p* < 0.001, uncorrected) posttest gray matter volume increase in the right cingulate gyrus (BA24) (Figure 3B) while the CT group showed a posttest increase (*p* < 0.001, uncorrected) in the left inferior frontal gyrus (BA9) and right superior frontal gyrus (BA6) (Figure 3C).

**Table 4:** **Within-group frontal ROI gray matter volume increases between pretest and posttest for each group separately**.

**Group**	**Region**	**Brodmann area**	**MNI coordinates**			***Z*-statistic**	***p*-value**	**Cluster size**
			***X***	***Y***	***Z***			
TX	Left middle frontal gyrus	10	−32	47	0	4.01	0.000	64
	Right frontal pole	11	16	60	−28	3.52	0.000	42
	Right anterior cingulate	32	23	43	8	3.35	0.000	9
DTC	Right cingulate gyrus	24	19	4	30	3.87	0.000	56
CT	Left inferior frontal gyrus	9	−45	6	18	3.64	0.000	36
	Right superior frontal gyrus	6	12	18	66	3.27	0.001	12

*Results are shown for changes at the p < 0.001 level (uncorrected). Note: TX, Alert-treated FASD group; DTC, delayed-treatment control FASD group; CT, typically developing control group*.

#### Hypothesis 3

**Figure 4:**
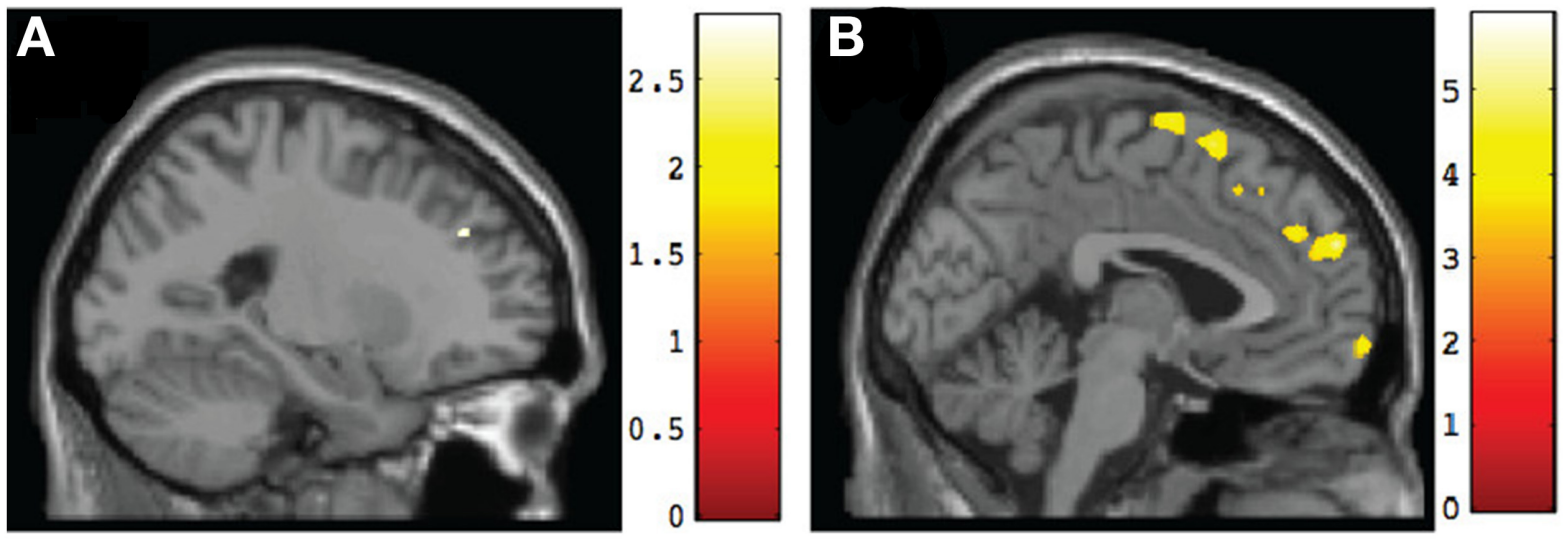
**Sagittal views of regions involving significant differences in gray matter volume at post-intervention. (A)** TX vs. DTC group, left superior frontal gyrus (BA9) [−23, 38, 32], *p* < 0.005, uncorrected; **(B)** CT vs. TX group, left superior frontal gyrus (BA9) [−2, 58, 27], *p* < 0.001, uncorrected.

Results of pairwise comparisons of posttest results using an ROI approach are presented in Table 5 (for regions involving large size clusters; remaining regions are presented in Supplementary Table 2). With the FDR correction applied, no significant between-group differences were found. However, when we used uncorrected data, the TX group indicated a marginally significantly larger (*p* < 0.005) gray matter volume than the DTC group in the left superior frontal gyrus (BA9) (Figure 4A), which was similar to a region that was initially larger at pretest in CT than the combined FASD group (i.e., the left superior frontal gyrus, BA8). However, multiple regions still remained significantly larger (*p* < 0.001, uncorrected) at posttest in CT than the TX group, including the left superior frontal gyrus (BA9) (Figure 4B).

**Table 5:** **Results of between-group pairwise posttest comparisons using frontal ROI masks**.

**Group**	**Region**	**Brodmann Area**	**MNI coordinates**			***Z*-statistic**	***p*-value**	**Cluster size**
			***X***	***Y***	***Z***			
TX > DTC	Left superior frontal gyrus	9	−23	38	32	2.60	0.005	29
CT > TX	Right superior frontal gyrus	6	3	15	62	4.68	0.000	669
	Right medial frontal gyrus	6	5	−7	69	4.25	0.000	1392
	Left superior frontal gyrus	9	−2	58	27	4.23	0.000	711
	Left cingulate gyrus	24	−13	2	43	3.78	0.000	324
	Left medial frontal gyrus	8	−4	27	45	3.62	0.000	201

*Findings indicate greater increases in gray matter volumes for TX compared with DTC group, and CT compared with TX group. For CT > TX, only clusters larger than 200 voxels are listed. Note: TX, Alert-treated FASD group; DTC, delayed-treatment control FASD group; CT, typically developing control group*.

#### Structure-function correlations

To explore relationships in the TX group between changes in neuroanatomy and changes in behavior, we performed simple correlations between changes in frontal ROI gray matter and changes in emotion regulation or inhibitory control between pretest and posttest sessions. These results are presented in Table 6. For the BRIEF Emotional Control scale, regions showing associations between behavioral improvement and increased gray matter volumes were the left medial frontal gyrus (BA10) and right inferior frontal gyrus (BA47). Similarly, regions showing a positive association between the TX group's degree of improvement on the NEPSY-II Inhibition subtest and the size of their gray matter increase were the bilateral superior frontal gyrus (BA10, BA8) and right middle frontal gyrus (BA6).

**Table 6:** **Regions showing post-intervention gray matter volume increases that were correlated with behavioral improvements in TX group**.

**Measure**	**Region**	**Brodmann Area**	**MNI coordinates**			***z*-statistic**	***p*-value**	**Cluster Size**
			***X***	***Y***	***Z***			
BRIEF	Left medial frontal gyrus	10	−11	50	16	3.79	0.000	121
	Right inferior frontal gyrus	47	44	36	−1	3.25	0.001	9
NEPSY-II	Right superior frontal gyrus	10	17	60	26	3.49	0.000	64
	Left superior frontal gyrus	8	−9	42	57	3.41	0.000	34
	Right middle frontal gyrus	6	41	7	63	3.38	0.000	41

## Discussion

This study represents one of the first of its kind, if not the first, to identify the neuroplastic changes that follow a relatively brief intervention targeting specific areas of weakness in children with FASD. Three hypotheses were tested: (1) at baseline, children with FASD would show less gray matter than controls in regions supporting EF (including self-regulation); (2) only the TX group would show gray matter volume changes between sessions in regions related to EF; and (3) at posttest, the neuroanatomy of the treated FASD group would closely resemble controls, whereas the neuroanatomy of the untreated FASD group (i.e., DTC) would remain unchanged. A supplementary goal was to explore whether the observed changes in brain anatomy in the TX group would correlate with changes in behavior and cognition following therapy.

Current findings provide only partial support for these hypotheses. While controlling for multiple comparisons, we did not find any significant changes among groups. However, uncorrected results revealed significant changes in gray matter volume. Specifically, we found that children with FASD at baseline (pretest) had less gray matter than controls in multiple regions relevant for EF. Additionally, we found that those children with FASD who received treatment subsequently demonstrated changes in critical regions for self-regulation, unlike children assigned to the waitlist condition who received treatment only on study completion, who showed only modest growth in one related area. At posttest, the TX group showed a modest increase in gray matter relative to DTC in a region that initially differentiated FASD and CT groups; however, the TX group still differed considerably from CT, contrary to predictions. Finally, we observed associations between several of the frontal lobe regions showing changes after Alert and improved emotional and inhibitory control.

Regarding our first hypothesis, we observed children in the typically developing control group demonstrated larger gray matter volumes than children with FASD in the left superior frontal gyrus (BA8) and bilateral medial frontal gyrus (BA8, BA32), which are critical regions for response inhibition (Lane et al., 1998; Norman et al., 2009). These findings confirm previous findings that PAE subsequently leads to reduced brain growth in frontal regions (Spadoni et al., 2007) and asymmetry in cortical surface gray matter (Sowell et al., 2002).

Our second hypothesis concerned how each group changed over time and whether this differed for those who received the intervention or not. We found that the TX group after Alert evinced increased gray matter in several frontal lobe regions critical for emotional or inhibitory control. These included the left middle frontal gyrus (BA10), which is crucial for response inhibition during go/no-go decisions (Simmonds et al., 2008), the right frontal pole (BA11), associated with outcome monitoring (Tsujimoto et al., 2011), and the right anterior cingulate (BA32), which is activated when suppressing emotional responses (Levesque et al., 2004) and also during self-inhibition (Beauregard et al., 2001). Given that difficulties in response inhibition and emotion regulation represent core impairments in children with FASD, our findings suggest that Alert may indeed serve to increase gray matter in these key brain regions. In other words, Alert was beneficial at the endophenotypic level. However, we are cautious with our interpretation of these data, as they are based on uncorrected data.

Unexpectedly, however, we also observed brain changes in both the DTC and CT groups. The DTC group showed increased size of the right cingulate gyrus (BA24), which is important for reward anticipation and modulation of emotions. Although it is not readily clear why repeat scanning may contribute to this change, it may reflect the effect of revisiting the hospital and anticipation of treatment still to come. Nevertheless, given this finding is based on uncorrected data, as mentioned above, it also may reflect a type 1 error. The CT group by contrast showed increased size of the left inferior frontal gyrus (BA9) and right superior frontal gyrus (BA6) between sessions. The left inferior frontal gyrus is important for language production and aspects of inhibitory control while the right superior frontal gyrus is important for self-awareness. It should be noted that as part of the larger study to which the current study belonged, all children were also assessed at both pretest and posttest with an inhibitory control paradigm. It is possible that the changes we observed in gray matter volumes of the CT group were related to this co-occurring experience, as well as to the usual growth that takes place over a 4-month period (Giedd et al., 1999). Again, alternatively, these changes may be spurious as they are based on uncorrected results. That the regions of change during the interval without therapy differed between DTC and CT groups may be indicative of differential growth in the two groups in regions that are important for self-regulation.

Hypothesis 3 predicted that after Alert, the TX group would resemble more closely the CT than the DTC group. Our findings generally did not support this hypothesis. We observed the CT group continued to show substantially larger gray matter volumes bilaterally throughout the medial and superior frontal lobes. However, we did observe that relative to the DTC group, the TX group did show a marginal gain in gray matter within the left superior frontal gyrus (BA9), which is implicated in intentional emotional control (Levesque et al., 2004). The continued differences between TX and CT groups are not surprising and suggest that the impact of PAE on the developing brain cannot be fully mediated by a brief 12-week intervention. This observation is supported by the behavioral evidence showing that although the TX did improve significantly following Alert training, their scores were still in the clinical range on the BRIEF Emotional Control scale (albeit far better than the DTC group) and moved to just within the normal range of the NEPSY-II Inhibition subtest (vs. above the normal range mean in CT).

Importantly, we found that several of the regions showing increased gray matter in the TX group were correlated with post-intervention improvements in neuropsychological outcome. Specifically, improved scores on the BRIEF Emotion Regulation scale were correlated with increased gray matter in the left medial frontal gyrus (BA10) and the right inferior frontal gyrus (BA47). These regions are notably important for emotion reappraisal (Levesque et al., 2004) and response suppression (Beauregard et al., 2001), respectively, which are both aspects of emotion regulation. Similarly improvements on the NEPSY-II Inhibition subtest were associated with increases in gray matter volumes of the bilateral superior frontal gyrus (BA10, BA8), which is implicated in self-inhibition (Norman et al., 2009), and the right middle frontal gyrus (BA6), which is implicated in response inhibition during a no-go task (Simmonds et al., 2008).

Studies of children with FASD using other neuroimaging modalities have found similar results. Fryer and colleagues, for example, observed on functional MRI that relative to controls, children with PAE on a go/no-go paradigm had increased bilateral BOLD activation in the middle frontal gyrus when they withheld responses (Fryer et al., 2007). This finding overlaps with our observation that the TX group showed growth in the left middle frontal gyrus (BA10) following treatment. A greater effect in the left hemisphere, as opposed to a bilateral effect, may reflect the fact that Alert was verbally-based and thus led to growth in left hemispheric regions primarily.

In animal models, it is shown that increased gray matter reflects synaptogenesis. For example, Biedermann et al. (2012) showed that increased gray matter was due to growth in neuronal dendrites and spines reflecting both new connections and the strengthening of existing ones (Holtmaat et al., 2006; Yasumatsu et al., 2008). Indeed, a study of adult rodents prenatally exposed to ethanol who were given an exercise program indicated increased neurogenesis within the hippocampus (Redila et al., 2006), signifying interventions can facilitate neuroplastic change in brains originally damaged by PAE. Our findings currently provide support for this possibility in humans, since we observed that the brain regions showing the most growth in children with FASD who received treatment were the ones that support the functions that Alert targets, namely response inhibition and emotional control.

### Strengths and limitations

Our study has several noteworthy strengths. First, our FASD sample primarily consisted of children who had received a clinical diagnosis along the FASD spectrum or had known confirmed heavy exposure to alcohol as the primary exposure *in utero*. Also, our sample spanned a relatively narrow age range compared with similar neuroplasticity studies in other populations (Lazaro et al., 2009; Huyser et al., 2013). Second, we used an empirically validated treatment for children with FASD (Wells et al., 2012). Third, we used a recognized technique to monitor structural brain changes over time. Finally, we included an FASD control group who were similarly followed but only received treatment upon study completion.

However, our study also had a number of important limitations regarding other aspects of our sample, as well as our analytic approach. Regarding sample characteristics, first and foremost, it should be noted that our final sample size contributing to analyzable MRI scans was relatively small for the types of analyses performed on the neuroimaging data. Nevertheless, it should be noted that we were constrained by the complexity of recruitment and family commitment leading to a large refusal rate as well as some sample loss during the study itself. Even though the population rates of FASD may be relatively high (May et al., 2014), it should be noted that our cases were all heavily exposed, severely affected, and also spanned a relatively narrow age range. Also, given that some cases were from foster families with a number of similarly difficult cases, the need for weekly commitment to treatment and extensive testing before and after treatment was beyond the limits of some families. Some families were unwilling to undergo randomization and sought immediate treatment elsewhere given the seriousness of the child's problems. Technical difficulties and the wearing of braces during the 4-month period of the study also led to sample reduction of children already entered into the study.

Another shortcoming pertaining to our sample is we did not assess for their stage of pubertal development given that neurodevelopment peaks sooner than in females than males (Giorgio et al., 2010; Raznahan et al., 2011) and the TX group unexpectedly had fewer females than the DTC group. Likewise, our sample spanned an age period when significant brain changes normally occur that reflect both increased and decreased size of gray matter (Peper et al., 2009; Giorgio et al., 2010). As a consequence, our sample's age, despite being ideal for Alert treatment and scanning, may have led to attenuation of findings given some children would be on an upward trajectory and others on a downward. Moreover, effects may differ for males and females (Peper et al., 2009) and given our small sample size, we were unable to perform comparisons separately by sex. Our TX group also by chance included more children with pFAS and had lower IQs than the DTC group. Thus, our findings might have been more robust had the two FASD groups been more similar.

Several design features also warrant mention. First, our approach cannot be considered a true randomized trial since we allowed sibling pairs to be assigned to the same treatment condition, for family convenience and to ensure participation and one family assigned to the TX group was moved to DTC due to scheduling conflicts. Also, the period of elapsed time between MRI sessions differed among the groups and was longest in TX. Thus, it is possible that some effects could have reflected a longer period of normal growth in this group vs. the others, especially CT, whose interval was shortest. Although the longer interval in TX was due to unanticipated delays in completing the 12 weeks of therapy, it should be noted that the booking times on the MRI for the other groups were established at the initial session (to ensure scanner availability), whereas the posttest MRI booking for the TX group could not be done until the therapy was finished. It is also possible that part of the delay reflected MRI availability on short notice. While ideally, we would have yoked cases from each group for posttest scanning sessions, this could not be done. There is no ready explanation as to why the interval was slightly longer in DTC than CT groups.

Regarding our analytic approach, the consequence of our small sample size and not perfectly balanced groups may have led to our inability to find effects when correction for multiple comparisons across the brain was applied (type 2 statistical error). Alternatively, some of our findings based on uncorrected data may represent a false-positive or type 1 statistical error given the large number of comparisons that were performed. Hopefully, by adopting a *p-*value less than 0.001, such errors only occur 1 in 1000 times. Secondly, although we used a frontal mask for our ROI analysis, due to this population's noted deficits in EF, the mask we chose, covering the entire frontal lobe region, was relatively large and still allowed for a large number of voxel comparisons.

Finally, we examined structure-function correlations only in the TX group, as our goal was to discern how treatment-related brain changes affected outcome in children with FASD. Our not having a comparable set of correlations for the other two control groups did not allow us to examine placebo-type or developmental effects on behavioral outcome.

Clearly, future studies need to replicate our work in a much larger sample of children with FASD, which will likely necessitate a collaborative enterprise among multiple centers.

## Conclusions

This study examining structural brain changes associated with the Alert Program for Self-Regulation in children with FASD is the first to identify the neuroanatomical changes following treatment for their core deficit in executive functioning. Our findings showing Alert succeeds in increasing cortical gray matter in children with FASD, particularly in regions underlying response inhibition, outcome monitoring, and emotion regulation signify it is possible to ameliorate, at an endophenotypic level, some of the brain damage caused by PAE. Thus, our research offers preliminary hope for improving functional outcome in children with FASD.

## Author contributions

DWS analyzed and interpreted the data and wrote and revised the manuscript. JS acquired and analyzed the data and revised the manuscript. KN conceptualized the study and acquired data. SS helped conceptualize the study and acquire data. JR conceptualized the study, was responsible for funding and management of the project, and co-wrote the manuscript and its revision.

### Conflict of interest statement

The authors declare that the research was conducted in the absence of any commercial or financial relationships that could be construed as a potential conflict of interest.
