# Supplementary material for: Self-regulation therapy increases frontal gray matter in children with fetal alcohol spectrum disorder: evaluation by voxel-based morphometry
Source: Front Hum Neurosci. 2015 Mar 4;9:108. doi: 10.3389/fnhum.2015.00108 (PMC4349084; doi:10.3389/fnhum.2015.00108)
Supplement: Supplementary file 1 [file Table1.DOCX]

**SUPPLEMENTARY MATERIAL**

**Self-regulation therapy increases frontal gray matter in children with fetal alcohol spectrum disorder: evaluation by voxel-based morphometry**

Debra W. Soh^1^, Jovanka Skocic^2^, Kelly Nash^2,3^, Sara Stevens^2,4^, Gary R. Turner^1^,

Joanne Rovet^2,4,5^ *

^1^ Department of Psychology, York University, Toronto, ON, Canada

^2^ Neurosciences and Mental Health Program, The Hospital for Sick Children,

Toronto, ON, Canada

^3^ The Ontario Institute of Studies in Education, University of Toronto, ON, Canada

^4^ Department of Psychology, University of Toronto, ON, Canada

^5^ Department of Pediatrics, University of Toronto, ON, Canada

*** Correspondence:** Joanne Rovet, Ph.D., Peter Gilgan Centre for Research & Learning, The Hospital for Sick Children, 686 Bay Street, Toronto, ON, Canada, M5G 0A4, Phone: 416-813-8283, Fax: 416-813-8839, Email: joanne.rovet@sickkids.ca

**Supplementary Tables**

**Supplementary Table 1 | Significant (uncorrected) pairwise comparisons between FASD-combined and CT groups using a whole-brain analysis. Findings indicate where FASD group showed larger gray matter volumes than CT group, and where CT group showed larger gray matter volumes than FASD group, for clusters smaller than 200 voxels.**

| Group | Region | Brodmann Area | MNI Coordinates  X Y Z | | | *Z*-  statistic | *p*-  value | Cluster Size |
| --- | --- | --- | --- | --- | --- | --- | --- | --- |
| FASD > CT | Right lingual gyrus |  | 27 | -70 | -3 | 3.69 | .000 | 163 |
| CT > FASD | Left paracentral lobule |  | -1 | -15 | 49 | 3.66 | .000 | 170 |
|  | Left superior frontal gyrus | 6 | -12 | 12 | 69 | 3.59 | .000 | 133 |
|  | Left postcentral gyrus |  | -50 | -14 | 54 | 3.50 | .000 | 59 |
|  | Left precentral gyrus | 4 | -40 | -17 | 55 | 3.39 | .000 | 53 |
|  | Left superior parietal lobule | 7 | -40 | -60 | 56 | 3.35 | .000 | 69 |
|  | Left superior frontal gyrus | 6 | -3 | 5 | 64 | 3.24 | .001 | 18 |
|  | Left inferior frontal gyrus | 44 | -59 | 20 | 14 | 3.22 | .001 | 23 |
|  | Left inferior frontal gyrus |  | -54 | 28 | 20 | 3.22 | .001 | 17 |
|  | Right superior frontal gyrus | 6 | 26 | -12 | 74 | 3.18 | .001 | 8 |
|  | Left medial frontal gyrus | 9 | -2 | 53 | 25 | 3.14 | .001 | 8 |
