# Supplementary material for: Self-regulation therapy increases frontal gray matter in children with fetal alcohol spectrum disorder: evaluation by voxel-based morphometry
Source: Front Hum Neurosci. 2015 Mar 4;9:108. doi: 10.3389/fnhum.2015.00108 (PMC4349084; doi:10.3389/fnhum.2015.00108)
Supplement: Supplementary file 2 [file Table2.DOCX]

**SUPPLEMENTARY MATERIAL**

**Self-regulation therapy increases frontal gray matter in children with fetal alcohol spectrum disorder: evaluation by voxel-based morphometry**

Debra W. Soh^1^, Jovanka Skocic^2^, Kelly Nash^2,3^, Sara Stevens^2,4^, Gary R. Turner^1^,

Joanne Rovet^2,4,5^ *

^1^ Department of Psychology, York University, Toronto, ON, Canada

^2^ Neurosciences and Mental Health Program, The Hospital for Sick Children,

Toronto, ON, Canada

^3^ The Ontario Institute of Studies in Education, University of Toronto, ON, Canada

^4^ Department of Psychology, University of Toronto, ON, Canada

^5^ Department of Pediatrics, University of Toronto, ON, Canada

*** Correspondence:** Joanne Rovet, Ph.D., Peter Gilgan Centre for Research & Learning, The Hospital for Sick Children, 686 Bay Street, Toronto, ON, Canada, M5G 0A4, Phone: 416-813-8283, Fax: 416-813-8839, Email: joanne.rovet@sickkids.ca

**Supplementary Tables**

**Supplementary Table 2 | Results of between-group pairwise posttest comparisons using frontal ROI masks. Findings indicate where CT group showed greater increases in gray matter volumes than TX group for clusters smaller than 200 voxels.**

| Group | Region | Brodmann Area | MNI Coordinates  X Y Z | | | *Z*-statistic | *p*-value | Cluster Size |
| --- | --- | --- | --- | --- | --- | --- | --- | --- |
| CT > TX | Right middle frontal gyrus |  | 28 | -7 | 68 | 3.61 | .000 | 63 |
|  | Right superior frontal gyrus | 6 | 15 | 15 | 65 | 3.57 | .000 | 103 |
|  | Right medial frontal gyrus | 8 | 6 | 25 | 44 | 3.51 | .000 | 76 |
|  | Left middle frontal gyrus | 10 | -26 | 61 | -8 | 3.28 | .001 | 19 |
|  | Left superior temporal gyrus | 38 | -32 | 25 | -33 | 3.28 | .001 | 25 |
|  | Right inferior frontal gyrus | 47 | 34 | 36 | -23 | 3.23 | .001 | 16 |
